# Identification of 2-Aminothiazole-4-Carboxylate Derivatives Active against *Mycobacterium tuberculosis* H_37_R_v_ and the β-Ketoacyl-ACP Synthase mtFabH

**DOI:** 10.1371/journal.pone.0005617

**Published:** 2009-05-19

**Authors:** Qosay Al-Balas, Nahoum G. Anthony, Bilal Al-Jaidi, Amani Alnimr, Grainne Abbott, Alistair K. Brown, Rebecca C. Taylor, Gurdyal S. Besra, Timothy D. McHugh, Stephen H. Gillespie, Blair F. Johnston, Simon P. Mackay, Geoffrey D. Coxon

**Affiliations:** 1 Strathclyde Institute of Pharmacy and Biomedical Sciences, University of Strathclyde, Glasgow, United Kingdom; 2 Department of Infection, University College London, London, United Kingdom; 3 Strathclyde Innovations in Drug Research, Glasgow, United Kingdom; 4 School of Biosciences, University of Birmingham, Edgebaston, Birmingham, United Kingdom; University of Sydney, Australia

## Abstract

**Background:**

Tuberculosis (TB) is a disease which kills two million people every year and infects approximately over one-third of the world's population. The difficulty in managing tuberculosis is the prolonged treatment duration, the emergence of drug resistance and co-infection with HIV/AIDS. Tuberculosis control requires new drugs that act at novel drug targets to help combat resistant forms of *Mycobacterium tuberculosis* and reduce treatment duration.

**Methodology/Principal Findings:**

Our approach was to modify the naturally occurring and synthetically challenging antibiotic thiolactomycin (TLM) to the more tractable 2-aminothiazole-4-carboxylate scaffold to generate compounds that mimic TLM's novel mode of action. We report here the identification of a series of compounds possessing excellent activity against *M. tuberculosis* H_37_R_v_ and, dissociatively, against the β-ketoacyl synthase enzyme mtFabH which is targeted by TLM. Specifically, methyl 2-amino-5-benzylthiazole-4-carboxylate was found to inhibit *M. tuberculosis* H_37_R_v_ with an MIC of 0.06 µg/ml (240 nM), but showed no activity against mtFabH, whereas methyl 2-(2-bromoacetamido)-5-(3-chlorophenyl)thiazole-4-carboxylate inhibited mtFabH with an IC_50_ of 0.95±0.05 µg/ml (2.43±0.13 µM) but was not active against the whole cell organism.

**Conclusions/Significance:**

These findings clearly identify the 2-aminothiazole-4-carboxylate scaffold as a promising new template towards the discovery of a new class of anti-tubercular agents.

## Introduction

The disease tuberculosis (TB), once considered eradicated, has again become a major global health concern. *M. tuberculosis*, the causative organism, produces a chronic infection in the lungs that can become disseminated. Over the past decade, at least 30 million individuals have died from the disease and estimates indicate that one-third of the world's population is infected with latent or persistent *M. tuberculosis*. Moreover, globally more than 8 million people develop active TB every year, and if trends continue there will be a total of 36 million disease-related deaths by the year 2020 [1], [2].

The resurgence in the disease is caused by an inadequate and extended chemotherapy that relies on drugs developed in the mid-twentieth century. The associated poor patient compliance and emergence of drug resistant forms of TB, coupled with a strong epidemiological co-existence with HIV/AIDS highlights the fundamental need for new, more effective drugs to treat the disease [3]–[5].

The mycobacterial cell wall of *M. tuberculosis* is rich with many unique key structural components that are necessary for the mycobacteria to survive and grow within the human host, and has long been a target for anti-TB drug development. Essential to the cell wall are the mycolic acids, which are high molecular weight 2-alkyl, 3-hydroxy fatty acids that exist in several forms of differing chemical functionality. Indeed, the first line anti-tubercular drug isoniazid (INH) works by inhibiting their biosynthesis. The complete sequencing of the TB genome [6] has revealed significant biochemical and genetic insight into mycolic acid biosynthesis that will aid the search for new druggable targets. These unique lipids are biosynthesised by both fatty acid synthase enzyme systems I and II (FAS I and FAS II) to produce C_56–64_ meromycolic acids and the C_26_ α-branch [7], [8] after a series of biostransformations [9], [10].

**Figure 1:**
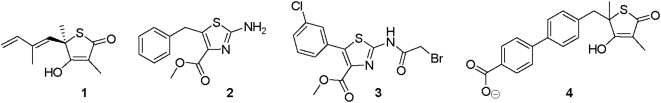
The chemical structures of thiolactomycin 1, its analogue 4, and inhibitors 2 and 3.

The naturally occurring antibiotic thiolactomycin **1** (TLM, figure 1) [11]–[13] primarily acts by inhibiting the FAS-II β-ketoacyl-ACP synthase condensing enzymes, halting mycolic acid biosynthesis and subsequently to *M. tuberculosis* cell death [14]–[17]. TLM is also orally available and non-toxic in the mouse model, which makes it an attractive compound for development. Conversely, the chemical scaffold of TLM possesses a chiral centre at the 5-position which makes the synthesis of series of TLM analogues lengthy and costly, and complicates the optimisation process. Such factors need to be considered carefully when developing economically viable drugs for developing countries.

This issue of synthetic tractability has focused researchers' efforts towards the synthesis of either racemic analogues or derivatives that contain simple modifications, and has yielded limited improvements in activity against *M. tuberculosis* and modest activity against mtFabH [18]–[22]. We have focussed on identifying alternative, easily accessible 5-membered ring isosteres to generate large compound libraries targeted against the condensing enzyme mtFabH and *M. tuberculosis*. Herein, we demonstrate that 2-aminothiazole-4-carboxylate offers a promising template for the development of new anti-tubercular agents, and report the design and synthesis of methyl 2-amino-5-benzylthiazole-4-carboxylate **2** as our most potent inhibitor of *M. tuberculosis H_37_R_v_*, with an MIC of 0.06 µg/ml (0.24 µM), which is more effective than both TLM and INH (MICs of 13 µg/ml (62.5 µM) [18] and 0.25 µg/ml (1.8 µM) [19] respectively (figure 1). We also show that methyl 2-(2-bromoacetamido)-5-(3-chlorophenyl)thiazole-4-carboxylate **3** inhibits mtFabH with an IC_50_ of 0.95 µg/ml (2.43 µM), which compares well with TLM **1** and its most potent analogue **4** (IC_50_ values of 16 µg/ml (75 µM) and 1.1 µg/ml (3.0 µM) respectively [22] (figure 1).

## Results and Discussion

### Ligand design

The initial focus of our ligand design was based on the active site geometry and mechanism of action of the target enzyme mtFabH, a homodimer (PDB: 1M1M) that converts C_12–20_ acyl-CoA substrates to the corresponding β-ketoacyl-AcpM product after reaction with mal-AcpM in a two step process [23]. At the molecular level, the acyl-CoA substrate enters an “L” shaped binding pocket consisting of a lateral and longitudinal channel, with the active site catalytic triad of Cys112-His244-Asn274 located at the junction. Transacylation of the Cys122 residue occurs when the adjacent His244 deprotonates the thiol group (directly or *via* a molecule of water, as postulated by Brown *et al.* [23]) to generate a thiolate nucleophile that attacks the carbonyl group of the acyl chain occupying the longitudinal channel, and releases CoA-SH from the lateral channel through which the substrate entered. The second substrate, mal-AcpM, then enters the lateral channel, and is decarboxylated by the catalytic residues His244 and Asn274, and condensed with the thioester formed at Cys112 to generate the β-ketoacyl-AcpM, which also dissociates via the lateral channel. Recently, Sachdeva *et al.* have postulated that the overall reaction may occur simultaneously in both active sites of the dimer [24].

As no inhibitor-mtFabH co-crystal structures have been solved to date, we investigated the binding pattern of TLM with the closely related analogue ecFabB from *E.coli* [25]. TLM reversibly inhibits ecFabB by forming a number of non-covalent interactions: the methyl group at carbon 3 of TLM is positioned in a hydrophobic pocket defined by residues Phe229 and Phe392; the 5-isoprenoid moiety is wedged between two peptide bonds - from above by residues Val271 and Phe272 and from below by Gly391 and Phe392, which are important for specificity [23]; the carbonyl oxygen forms two H-bonds with the two histidines in the active site; the 4-hydroxyl group H-bonds to the carbonyl oxygen of Val270 and the amide NH of Gly305 through a lattice of three water molecules; and the sulfur is adjacent to the active site Cys residue, although without any obvious interaction. The strong H-bonding between the active site His residues of ecFabB and the TLM carbonyl group is believed to be crucial for effective inhibitory activity against this enzyme. Based on this analysis, we postulated that the closely related active site of mtFabH could be expected to form an equivalent H-bonding network with TLM, and any new isosteric scaffold would need to maintain many of these important interactions. The equivalent condensing enzyme from *E. coli* (ecFabH) is also closely related to mtFabH, and has been co-crystalised with the very potent inhibitor 2-hydroxy-6-(3-phenoxy-4-phenyl-benzamido) benzoic acid [26]. This complex revealed an important role for a carboxylic acid moiety in the ligand as it forms specific interactions in the active site with the His250 residue from ecFabH [26]. We considered inclusion of this moiety to be important for our inhibitors and proposed the achiral 2-amino thiazole-5-carboxylate scaffold as an alternative to the TLM substructure to combine pharmacodynamic potency with essential pharmacokinetic considerations such as solubility. Finally, synthetic tractability and subsequent diverse library generation were considered possible using the simple procedure described by Barton *et al.* [27].

**Figure 2:**
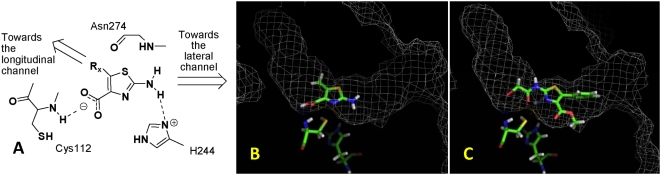
The modeling studies of the 2-aminothiazole-4-carboxylate analogues with mtFabH. (A) The hypothetical template of the 2-aminothiazole-4-carboxylates for mtFabH inhibitor development. This illustrates the key H-bonding interactions with the catalytic triad amino acid residues. (B) The binding pose of methyl 2-amino-5-methylthiazole-4-carboxylate in the active site of mtFabH showing the NH_2_ group proximal to His 244 and directed towards the lateral channel, with the 5-methyl group directed towards the longitudinal channel. (C) The binding pose of methyl 2-(2-bromoacetamido)-5-(3-chlorophenyl)thiazole-4-carboxylate with the bromomethylene portion in the vicinity of the Cys112 thiol group.

Using GOLD, docking studies were performed to investigate the poses for our scaffold in the mtFabH active site [28]. We observed that the carboxyl group of the thiazole ring forms H-bonds with the NH of Cys112, whilst the NH_2_ is proximal to and H-bonds with the imidazole ring of His244. This allowed us to generate a hypothetical template for the development of inhibitors of mtFabH (Figure 2A). Further docking studies were carried out with phenyl, *m*-chlorophenyl (figure 2B) and benzyl substituents in the 5-position in both the 4-methyl ester and free acid forms of the thiazoles. In these cases, the longitudinal channel appears to be the preferred residence for the 5-substituents, whilst the 2-amino group and potentially amide derivatives thereof could be accommodated in the lateral channel. We also noted that a 2-bromoacetamido substituent in this position would place the thiol group of Cys112 in a position to become alkylated *via* a nucleophilic S_N_2 reaction, and could lead to irreversible inhibition of the enzyme (figure 2C). Whilst such a strategy would normally be performed once a selective and relatively potent inhibitor had been found, we postulated that it would be reasonable at this early stage of inhibitor design to attempt this in order to establish if ligand inhibition was at all possible before addressing selectivity in later rounds of ligand optimisation. Based on this rationale, we prepared a series of thiazoles that included the substituents studied for evaluation against the enzyme and *M. tuberculosis*.

### Chemical Synthesis

**Figure 3:**
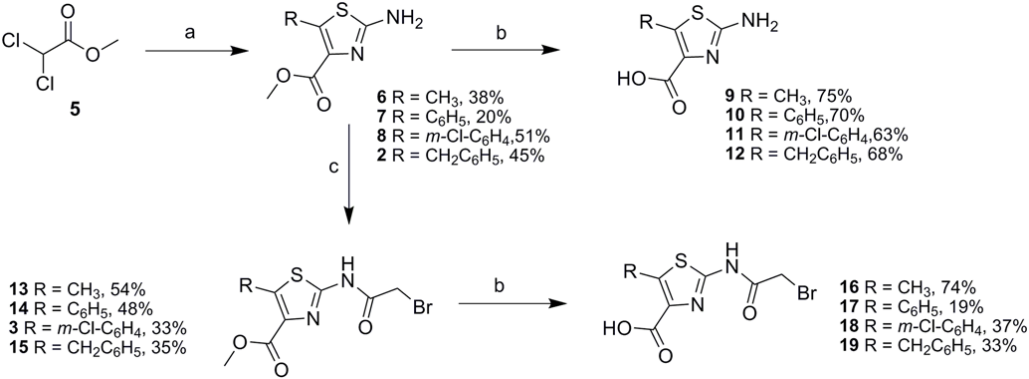
Synthesis of the 2-aminothiazole-4-carboxylate analogues.

Using the flexible synthetic procedure described by Barton *et al.* [27], the synthesis of the thiazole derivatives (figure 3) was achieved starting with the Darzens reaction between methyl dichloroacetate **5** and the appropriate aldehyde. This afforded a mixture of the α-chloro glycidic ester and β-chloro α-oxoester, which was extracted with diethylether and immediately reacted with thiourea dissolved in methanol to generate the methyl ester thiazoles **2**, **6–8**. To investigate whether a free carboxylic acid functionality would increase binding within the active site through facilitating electrostatic interactions proposed by the modeling studies, we hydrolysed the esters with 0.1 M sodium hydroxide solution followed by workup with dilute hydrochloric acid to generate compounds **9–12**. In order to generate 2-bromoacetamido analogues, the free amines **2**, **6–12** were reacted with 2-bromoacetylchloride in anhydrous THF at 0°C to afford the amides **3**, **13–15**. As described previously, hydrolysis with 0.1 M sodium hydroxide gave the corresponding carboxylic acids **16–19** for comparison.

### *In vitro* structure activity relationships

#### Inhibition of mtFabH

**Figure 4:**
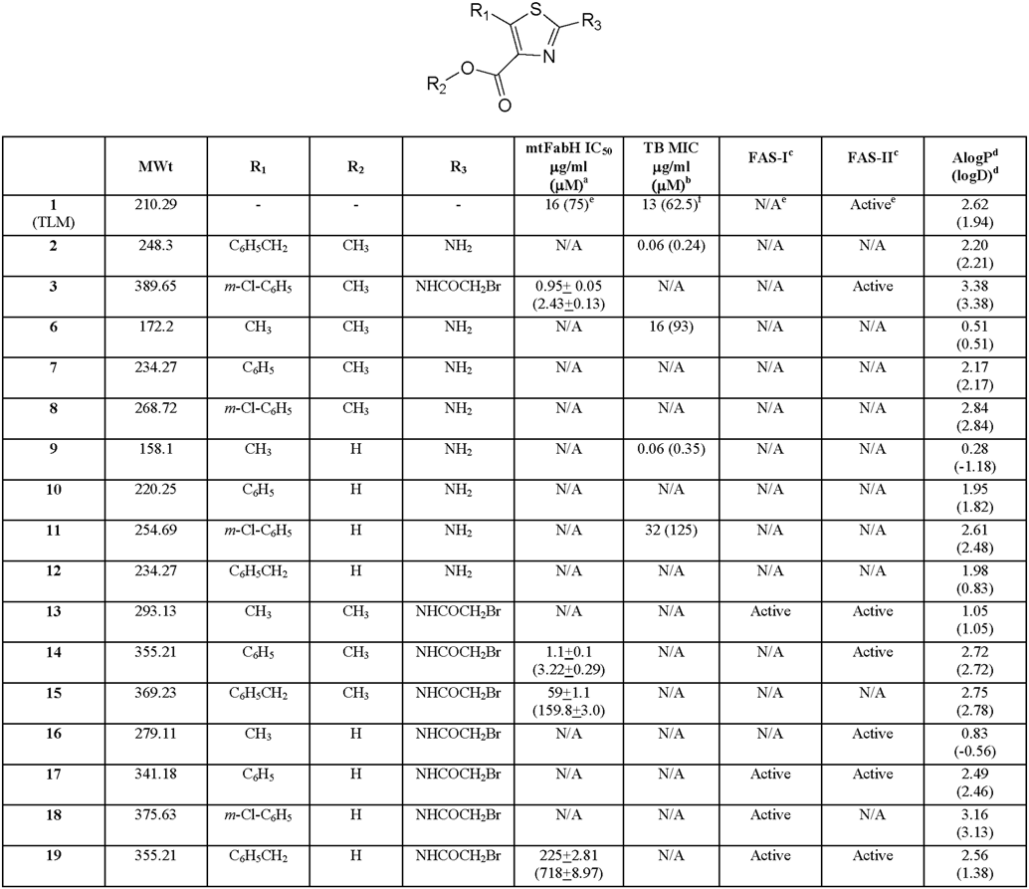
The *in vitro* activity and molecular properties of the 2-aminothiazole-4-carboxylates. ^a, b^ Compounds regarded as not active (N/A) if no inhibition is observed at 200 µg/ml. ^c^ FAS-I/II assay conducted at 200 µg/ml and compounds regarded as not active is <50% inhibition observed. ^d^ AlogP and logD calculated using Pipeline Pilot (SciTegic) software. ^e^From reference [18]. ^f^From reference [22].

The compounds were first assessed against the target enzyme mtFabH using the procedure developed by Brown *et al.* [23] (figure 4). Despite many of them not demonstrating any inhibitory activity at a concentration of 200 µg/ml, it was pleasing to see that the bromoacetamido analogues that were prepared to facilitate an S_N_^2^ type substitution between the ligand and the Cys112 residue, were active. The bromoacetamido esters **3**, **14** and **15** inhibited the enzyme with IC_50_ values of 0.95±0.05 (2.43±0.13 µM), 1.1±0.1 µg/ml (3.22±0.29 µM) and 59±1.1 µg/ml (159.8±3.0 µM) respectively, whilst the corresponding carboxylic acid **19** inhibited the enzyme at 225±2.81 µg/ml (718±8.97 µM). Interestingly, the ester **13** and carboxylic acids **16**, **17** and **18** failed to inhibit the enzyme.

It is clear that whilst the electrophilic bromomethyl substituent establishes activity against the enzyme, its effect is modified by different substituents at the 4- and 5- positions. Assuming that the inhibition observed involves reaction with the Cys112 residue, then the ligands must be situated in the vicinity of the catalytic triad. As the longitudinal and lateral tunnels are composed of lipophilic amino acid residues, we suggest that **13** and **16**, which possess methyl groups at position 5, are unable to maximize the hydrophobic interactions with these channels necessary to facilitate enzyme inhibition. However, inserting a phenyl group at position 5 and augmenting it with a m-Cl, as in **14** and **3** respectively, enables the appropriate hydrophobic interactions with the enzyme to occur and enables effective inhibition. The flexibility of the ligand also appears to be an important factor; the m-Cl phenyl carboxylic acid analogue **18** is inactive whereas its 5-benzyl counterpart **19**, which is less lipophilic, inhibits the enzyme with an IC_50_ of 225±2.81 µg/ml (718±8.97 µM). Comparison with its benzyl ester **15**, which inhibits mtFabH with an IC_50_ of 59±1.1 µg/ml (159.8±3.0 µM), suggests that both hydrophobicity and flexibility at the 5-position of the thiazole ring are instrumental in orientating the ligand to achieve effective inhibition. It is interesting that **13** fails to inhibit mtFabH as it appears that the free acids are weaker inhibitors than the esters. This may be accounted for by the molecule possessing a methyl group at position 5 which does not enable it to maximize the hydrophobic interactions necessary to bind to the enzyme. Conversely, such interactions may be permitted by the flexible benzyl substituent on the free acid **19** which enables weak inhibition.

#### Inhibition of *M. tuberculosis* H_37_R_v_

Whilst it was encouraging to find that a small number of compounds inhibited the target enzyme, it was important to know if this activity would lead to inhibition of the whole cell organism. From the data obtained it was clear that all of the bromoacetamido analogues failed to inhibit *M. tuberculosis* H_37_R_v_ (figure 4). We do not know, at this stage, whether these compounds did not inhibit the mycobacteria through an inability to access the target enzyme due to inappropriate physicochemical properties, or because the bromomethyl moiety was chemically or metabolically inactivated by the *Mycobacterium*.

In contrast to the 2-bromoacetamido analogues, four of the free amine compounds (**2**, **6**, **9** and **11**) inhibited *M. tuberculosis* H_37_R_v_ with MIC values of 0.06, 16, 0.06 and 32 µg/ml (0.24, 93, 0.35 and 125 µM) respectively, whilst the other analogues of this group showed no activity. Given that these compounds did not inhibit mtFabH, their mechanism of action must involve other targets within the organism.

Although these compounds exhibit excellent activity, in the absence of any recognisable trends in the series, it is difficult to ascertain clear structure activity relationships. The best activity was obtained with **2** with a benzyl group in the 5-position and a methyl ester in the 4-position, whereas the carboxylic acid analogue was shown to be inactive. The opposite observations were seen with the inactive methyl ester analogue **8** possessing an m-Cl phenyl group at the 5-position and the corresponding active acid **11**. A similar trend was observed for the 5-methyl analogues **9** and **6** with MIC values of 0.06 and 16 µg/ml (0.35 and 93 µM) respectively. We speculate that these observations may result from the compounds' ability to enter the cell. In all cases, the primary amine at the 2-position would be associated with a dissociation equilibrium at physiological pH which could penetrate cellular membranes in the unionized state. Compounds **9** and **11** on the other hand, with both carboxylic acid and amino substituents, would exist as zwitterions and would not normally be expected to penetrate the lipophilic cell wall. The uptake of these compounds could involve a cellular uptake mechanism, such as mycobacterial porins that the inactive zwitterionic compounds **10** and **12** are not substrates for. The inactivity of the 2-amino analogue **7** is more likely due to its inability to interact structurally with the target in the organism.

#### Specificity of the 2-amino thiazole-4-carboxylates

The molecules under investigation were designed to inhibit mtFabH in the mycobacterial FAS-II system, with selectivity over the related mammalian FAS-I system. To examine selectivity, the compounds were assessed using the procedures of Brown *et al.* [23] and Slayden *et al.* [13].

**Figure 5:**
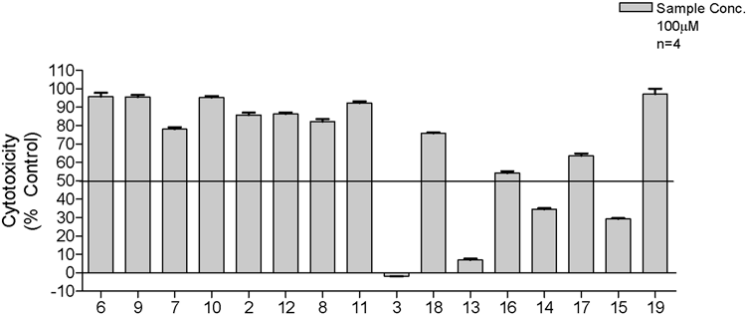
The cytotoxic effects of the compounds against HS-27 human fibroblast cells. The data indicates that only the carboxyl esters of the bromoacetamido analogues were toxic.

No inhibition of the FAS enzymes was observed, either as the acid or ester, when the 2-position was the free amine (figure 5). These data support the possibility that activity against *M. tuberculosis* H_37_R_v_ of compounds **2**, **6**, **9** and **11** involves a target other than mtFabH, or indeed the other FAS-II enzymes. However, with the exception of **15** all of the compounds possessing the bromoacetamido group at the 2-position showed activity against the FAS enzymes although no obvious trends were observed across this series. This is perhaps not surprising as these compounds may be inhibiting other enzymes in the FAS-II system or indeed at different sites in the multifunctional FAS-I complex. However, it was evident from the data that the phenyl **14** and m-Cl-phenyl **3** analogues were the only compounds active against mtFabH and selective against FAS-II. The mtFabH inhibitor **19** inhibited both FAS-I and FAS-II, whereas **16** inhibited only FAS-II and **18** FAS-I. Intriguingly, compound **15** failed to inhibit the FAS enzymes although inhibited mtFabH with an IC_50_ of 59±1.1 µg/ml (159.8±3.0 µM). While **15** may have shown activity against purified mtFabH, its inhibitory potential against mtFabH in the crude FAS-II assay may be difficult to detect as the related enzyme KasA (present in the crude reaction mix) has been shown to have FabH-type activity and could have had a compensatory effect [17].

Cytoxicity was investigated against human foreskin fibroblast HS-27 cells (Figure 5) to establish toxicity profiles for our compounds. The results indicated that none of the 2-amino analogues or the free carboxylic analogues of the bromoacetamido compounds showed significant cytotoxicity at a concentration of 100 µg/ml. Conversely, the carboxylic esters of the bromoacetamido compounds **3**, **13**, **14** and **15** all showed signs of significant cytotoxicity. We speculate that this may be due to the indiscriminate alkylation of essential cellular components rather than the increased ability of these esters to penetrate the cells over the carboxylic acids. This is supported by the fact that the non-cytotoxic acids **17**, **18** and **19** have comparable logD values to those of the cytotoxic esters **13**, **14** and **15** and thus possess similar physicochemical characteristics.

### Considerations for future development of the 2-aminothiazole-4-carboxylate scaffold

Our data clearly indicate that the 2-aminothiazole-4-carboxylate scaffold offers a promising new lead for further development. Methyl 2-amino-5-benzylthiazole-4-carboxylate **2** inhibits *M. tuberculosis* with an MIC of 0.06 µg/ml (240 nM) and is not cytoxic against HS-27 cells at 100 µg/ml concentrations. These compounds do not appear to inhibit the enzyme mtFabH, and it is important that their mode of action is elucidated for optimisation of the ligand to be carried out effectively. In doing so, care must be taken to retain the non-toxic and oral bioavailability properties of the molecule. However, whilst the zwitterionic, low molecular weight compound **9** has a structure that is highly hydrophilic, which has implications for both absorption and excretion by patients, this should be viewed in the context of INH, a successful and routinely administered anti-TB drug, which also has a low molecular weight and a logP value of −1.1.

## Materials and Methods

### Computational methods

#### Definition of active site

The active site determines the volume taken into consideration during the docking process and was defined as all protein atoms within 20 Å of the sulphur atom of Cys112.

#### Ligand and protein flexibility

All rotatable bonds of the ligand were randomized at the beginning of the GOLD docking and treated as completely flexible during the docking runs. Bond lengths, angles and torsions associated with non-rotatable bonds in the ligand were fixed in their initial configurations. The protein in GOLD is normally treated as rigid, with partial flexibility applied to hydroxyl protons and NH_3_^+^ groups, to allow rotation for hydrogen bond optimization.

#### GOLD docking

For each independent genetic algorithm (GA) run, a maximum number of 100,000 operations were performed on a population of 5 islands of 100 individuals. Operator weights for crossover, mutation and migration were set to 95, 95 and 10 respectively. Limits for van der Waals contacts and hydrogen bonds were assigned as 4.0 Å and 2.5 Å respectively and “GOLD Score” was chosen to assess the fitness of docked ligand-protein poses. Fifty poses were saved and visualized inside the active site to provide incite into potential binding patterns. Water molecules were removed from the pdb file and ligands were docked directly without any further preparation of the protein as recommended by the vendor.

### Experimental methods

#### Reagents and apparatus

Melting point determination was performed using a Stuart Scientific Melting Point SMP1 apparatus with degrees Celsius (°C) as the unit. Infra-red spectra were run on Jasco FT-IR-4200 ATR (Attenuated Total Reflection Mode) and Mattson Genesis Series FT-IR spectrometers with samples compressed with KBr into disks. Wavenumbers, (ν _max_) are expressed as cm^−1^. Proton nuclear magnetic resonance (^1^H NMR) and carbon (^13^C NMR) spectra were run on JEOL EX 270 (270 MHz), Bruker AMX-400 (400 MHz) and JEOL Lambda delta 400 (400 MHz) spectrometers. Chemical shifts are stated in parts per million (ppm) and multiplicity indicated as singlet (s), doublet (d), triplet (t), quartet (q), and multiplet (m). Coupling constants (J) are quoted in Hertz (Hz) and deuterated solvents specified for each of the compounds. High-resolution mass spectroscopy (MS) was obtained using Fourier transform electrospray ionisation (FTMS-ESI) and fast atom bombardment (3-nitrobenzyl alcohol matrix) (FAB-NOBA) ionizations on a JEOL JMS-700 Dual-sector High-resolution Mass Spectrometer. Mass to charge ratio (m/z) and relative abundance stated for molecular ion radicals is M^+^.. Unless otherwise stated, all reagents and solvents were obtained from commercial sources. Solvents were dried according to standard procedures when deemed necessary.

#### Synthesis of methyl 2-amino-5-benzylthiazole-4-carboxylate (2) as a general method for the synthesis of 2-amino-5-derivatized 4-carboxylate thiazoles (General procedure A)

Phenylcetaldehyde (1.6 g, 36 mmol, 1.0eq) was added to a stirring solution of methyl dichloroacetate 5 (5 g, 36 mmol, 1.0eq) dissolved in anhydrous ether (150 ml) at 0°C before adding NaOMe (1.65 g, 54 mmol, 1.5eq) dissolved in anhydrous MeOH (20 ml) dropwise over a period of 45 minutes. The mixture was allowed to stir for a further one hour at 0°C before adding brine (150 ml), extracting the organic phase with ether (150 ml), drying over MgSO_4_, and reducing *in vacuo*. To the crude residue was added thiourea (1.9 g, 36 mmol, 1.0eq) dissolved in MeOH (100 ml) and the mixture refluxed for 4 hours before concentrating *in vacuo* and neutralizing with concentrated ammonium hydroxide solution. The mixture was washed with dichloromethane (DCM) (3×, 50 ml), dried over anhydrous MgSO_4_, and purified by re-crystallization using chloroform and methanol (1∶3) to give **2** as a yellow powder (4.0 g, 44.9%). m.p 92–94°C; ^1^H NMR (270 MHz, DMSO-*d*_6_) δ 3.74 (s, 3H), 4.32 (s, 2H), 7.02 (s, 2H), 7.32 (m, 5H); ^13^C NMR (270 MHz, DMSO-*d*_6_) δ 31.7, 51.0, 126.1, 127.9, 128.8, 135.5, 136.4, 139.7, 162.3, 164.3; υ_max_ (cm^−1^): 3465, NH stretch, amine; 1711, C = O stretch; FAB/NOBA-MS calculated C_12_H_13_N_2_O_2_S (M+H) 249.0698, found 249.0696.

#### Synthesis of methyl 2-(2-bromoacetamido)-5-(3-chlorophenyl)thiazole-4-carboxylate (3) as a general procedure for the synthesis of the bromoacetamido derivatives (general procedure B)

Compound **8** (3.0 g, 17.4 mmol, 1.0eq) was added to a stirring solution of anhydrous tetrahydrofuran (THF) (100 ml) and triethylamine (TEA) (3.5 g, 34.8 mmol, 2.0eq) at 0°C before adding acetyl chloride (3.45 g, 17.4 mmol, 1.0eq) dropwise over a period of 30 minutes. The mixture was left to stir for a further 30 minutes at 0°C before allowed to warm to room temperature for one hour. THF was reduced *in vacuo* and the crude residue was re-dissolved in a mixture of DCM and water before the pH of the solution was adjusted to 3.0 using 0.1 M HCl. The mixture was washed with DCM (3×50 ml), dried over anhydrous MgSO_4_, and purified by re-crystallization using chloroform and methanol (1∶6) to give **3** as a white powder (1.4 g, 33.1%). m.p 218–220°C. ^1^H NMR (400 MHz, DMSO-*d*_6_) δ 3.80 (s, 3H), 4.11 (s, 2H), 7.36–7.39 (m, 3H), 7.47 (s, 1H); ^13^C NMR (400 MHz, DMSO-*d*_6_) δ 27.7, 52.2, 128.4–131.7, 134.2, 139.0, 156.0, 162.3, 164.9; υ_max_ (cm^−1^) 3216, NH stretch, 1699, C = O stretch, conjugated ester; 1684, C = O stretch, primary amide; FTMS-ESI calculated C_13_H_11_N_2_O_3_SClBr (M+H) 388.9362, found 388.9323.

#### Methyl 2-amino-5-methylthiazole-4-carboxylate (6)

The title compound was obtained as a pale yellow powder (1.7 g, 38.3%) using general procedure A. m.p 165–168°C. ^1^H NMR (270 MHz, DMSO-d_6_) δ 2.49 (s, 3H), 3.70 (s, 3H), 6.97 (s, 2H); ^13^C NMR (270 MHz, DMSO-d_6_) δ 12.7, 52.1, 135.2, 137.1, 164.3, 167.5; υ_max_ (cm^−1^) 3433, NH stretch, amine; 1688, C = O stretch, conjugated ester; FTMS-ESI calculated C_6_H_9_N_2_O_2_S (M+H) 173.0385, found 173.0379.

#### Methyl 2-amino-5-phenylthiazole-4-carboxylate (7)

The title compound was obtained as a pale yellow powder (1.7 g, 20.4%) using general procedure A. m.p 218–221°C. (Lit: 223°C) [28]. ^1^H NMR (270 MHz, DMSO-*d*_6_) δ 3.65 (s, 3H), 7.25 (s, 2H), 7.29–7.39 (m, 5H); ^13^C NMR (270 MHz, DMSO-*d*_6_) δ 128.35, 128.68, 129.88, 131.65, 131.98, 137.54, 164.19, 166.13; υ_max_ (cm^−1^) 3411, NH stretch, amine; 1696, C = O stretch, conjugated ester. FAB/NOBA-MS calculated C_11_H_11_N_2_O_2_S (M+H) 235.0541, found 235.0545.

#### Methyl 2-amino-5-(3-chlorophenyl)thiazole-4-carboxylate (8)

The title compound was obtained as a pale yellow powder (2.3 g, 50.7%) using general procedure A. mp 229–232°C. ^1^H NMR (400 MHz, DMSO-*d*_6_) δ 3.70 (s, 3H), 7.76 (m, 3H), 7.81 (s, 1H); ^13^C NMR (400 MHz, DMSO-*d*_6_) δ 52.0, 128.19, 128.8, 129.01, 129.95, 130.54, 132.68, 133.13, 136.1, 162.26, 166.1; υ_max_ (cm^−1^) 3417, NH stretch; 1699, C = O stretch; FAB/NOBA-MS calculated C_11_H_10_N_2_O_2_SCl (M+H) 269.0152, found 269.0163.

#### Synthesis of 2-amino-5-methylthiazole-4-carboxylic acid (9) as a general method for the hydrolysis of 2-amino-5-derivatized 4-carboxylic acid thiazoles (general procedure C)

Compound **6** (1.0 g, 6.3 mmol, 1.0eq) was added to a stirring solution of NaOH (150 ml, 85 mM) at 50–60°C over a period of 30 minutes before a clear solution formed, cooled and acidified with 1 M HCl to pH 3–4. A precipitate was formed and collected in a Buchner funnel, which was re-crystallized using methanol to give 9 (0.8 g, 75.0%). mp 320–324°C; ^1^H NMR (270 MHz, DMSO-d_6_) δ 2.49 (s, 3H), 6.97 (s, 2H); ^13^C NMR (270 MHz, DMSO-d_6_) δ 12.7, 135.2, 137.1, 164.3, 167.5; υ_max_ (cm^−1^) 3433, NH stretch, amine; 1688, C = O stretch, ester; 1688, COO stretch, carboxylic acid; FTMS-ESI calculated C_5_H_7_N_2_O_2_S (M+H) 159.0228, found 159.02138.

#### 2-amino-5-phenylthiazole-4-carboxylic acid (10)

The title compound was obtained as a white needle-like crystals (2.4 g, 70%) using general procedure C. mp 242–243°C; ^1^H NMR (400 MHz, DMSO-*d*_6_) δ 7.2 (bs, 1H), 7.35 (m, 4H), 12.5 (bs, 1H); ^13^C NMR (400 MHz, DMSO-*d*_6_) δ 128.35, 128.68, 129.88, 131.66, 131.98, 137.54, 164.2, 166.13; υ_max_ (cm^−1^) 3364, NH stretch, primary amine; 1701, C = O stretch, carboxylic acid; FTMS-ESI calculated C_10_H_9_N_2_O_2_S (M+H) 221.0385, found 221.0377.

#### 2-amino-5-(3-chlorophenyl)thiazole-4-carboxylic acid (11)

The title compound was obtained as a white powder (2.4 g, 63.3%) using general procedure C. mp 242–244°C; ^1^H NMR (400 MHz, DMSO-*d*_6_) δ 7.30–7.37 (m, 3H), 7.48 (s, 1H); ^13^C NMR (400 MHz, DMSO-*d*_6_) δ 128.2, 129.02, 131.4, 133.2, 134.0, 138.2, 163.9, 166.5; υ_max_ (cm^−1^) 3274, NH stretch, primary amine; 1684, C = O stretch, carboxylic acid; FAB/NOBA-MS calculated C_10_H_8_N_2_O_2_SCl (M+H) 254.9995, found 254.9982.

#### 2-amino-5-benzylthiazole-4-carboxylic acid (12)

The title compound was obtained as a yellow powder (0.6 g, 68.0%) using general procedure C. m.p 300–302°C; ^1^H NMR (400 MHz, DMSO-*d*_6_) δ 4.32 (s, 2H), 7.02 (s, 2H), 7.32 (m, 5H); ^13^C NMR (400 MHz, DMSO-*d*_6_) δ 32.6, 127.0, 128.8, 129.06, 136.4, 137.6, 140.9, 164.2, 165.1; υ_max_ (cm^−1^) 3465, NH stretch, amine; 1711, C = O stretch, carboxylic acid.; FAB/NOBA-MS calculated C_11_H_11_N_2_O_2_S (M+H) 235.0541, found 235.0529.

#### Methyl 2-(2-bromoacetamido)-5-methylthiazole-4-carboxylate (13)

The title compound was obtained as an off-white powder (2.6 g, 53.7%) using general procedure B. mp 220–222°C; ^1^H NMR (400 MHz, DMSO-*d*_6_) δ 2.62 (s, 3H), 3.79 (s, 3H), 4.12 (s, 2H), 12.8 (s, 1H); ^13^C NMR (400 MHz, DMSO-*d*_6_) δ 12.66, 28.7, 52.19, 135.9, 138.7, 153.7, 163.0, 166.0; υ_max_ (cm^−1^) 3235, NH stretch, primary amide; 1717, C = O stretch, conjugated ester; 1699, C = O stretch, primary amide; FTMS-ESI calculated (M+H) 292.9595, found 292.9582.

#### Methyl 2-(2-bromoacetamido)-5-phenylthiazole-4-carboxylate (14)

The title compound was obtained as a brown powder (0.8 g, 47.9%) using general procedure B. mp 218–220°C; ^1^H NMR (400 MHz, DMSO-*d*_6_) δ 3.69 (s, 3H), 4.16 (s, 2H), 7.48 (m, 5H), 13.08 (s, 1H); ^13^C NMR (400 MHz, DMSO-*d*_6_) δ 28.6, 52.3, 128.9–130.5, 135.1, 139.5, 155.6, 162.6, 166.4; υ_max_ (cm^−1^) 3230, NH stretch, primary amide; 1697, C = O stretch, conjugated ester; 1697, C = O stretch, primary amide; FAB/NOBA-MS calculated C_13_H_12_BrN_2_O_3_S (M+H) 354.9752, found 354.9752.

#### Methyl 5-benzyl-2-(2-bromoacetamido)thiazole-4-carboxylate (15)

The title compound was obtained as a brown powder (1.5 g, 34.5%) using general procedure B. mp 218–220°C; ^1^H NMR (400 MHz, DMSO-*d*_6_) δ 3.96 (s, 3H), 4.05 (s, 2H), 4.54 (s, 2H), 7.32 (m, 5H), 9.90 (s, 1H); ^13^C NMR (400 MHz, DMSO-*d*_6_) δ 28.6, 32.5, 52.3, 127.3, 129.11, 129.25, 135.6, 140.5, 143.2, 154.7, 163.5, 166.0; υ_max_ (cm^−1^) 3231, NH stretch, primary amide; 1701, C = O stretch, conjugated ester; 1701, C = O stretch, primary amide; FAB/NOBA-MS calculated C_14_H_14_N_2_O_3_SBr (M+H) 368.9908, found 368.9902.

#### 2-(2-Bromoacetamido)-5-methylthiazole-4-carboxylic acid (16)

The title compound was obtained as an off-white powder (0.7 g, 73.5%) using general procedure C. mp 218–220°C; ^1^H NMR (400 MHz, DMSO-*d*_6_) δ 2.65 (s, 3H), 4.11 (s, 2H), 12.8 (s, 1H); ^13^C NMR (400 MHz, DMSO-*d*_6_) δ 12.77, 28.82, 137.0, 138.5, 153.0, 164.0, 166.0; υ_max_ (cm^−1^) 3185, NH stretch, primary amide; 1699, C = O stretch, carboxylic acid; 1662, C = O stretch, primary amide. FTMS-ESI calculated C_7_H_8_BrN_2_O_3_S (M+H) 278.9439, found 278.9425.

#### 2-(2-Bromoacetamido)-5-phenylthiazole-4-carboxylic acid (17)

The title compound was obtained as a brown powder (0.1 g, 18.6%) using general procedure C. mp 208–210°C; ^1^H NMR (400 MHz, DMSO-*d*_6_) δ 4.16 (s, 2H), 7.41 (m, 5H); ^13^C NMR (400 MHz, DMSO-*d*_6_) δ 28.7, 128.8, 129.21, 130.31, 137.8, 139.1, 155.0, 164.0, 167.0; υ_max_ (cm^−1^) 3216, NH stretch, primary amide; 1675, C = O stretch, carboxylic acid; 1675, C = O stretch, primary amide. FAB/NOBA-MS calculated C_12_H_10_BrN_2_O_3_S (M+H) 340.9595, found 340.9600.

#### 2-(2-Bromoacetamido)-5-(3-chlorophenyl)thiazole-4-carboxylic acid (18)

The title compound was obtained as a white powder (0.3 g, 37.2%) using general procedure C. mp 228–230°C; ^1^H NMR (400 MHz, DMSO-*d*_6_) δ 4.17 (s, 2H), 7.46 (m, 3H), 7.61 (s, 1H); ^13^C NMR (400 MHz, DMSO-*d*_6_) δ 28.6, 114.98, 123.96, 128.46, 129.04, 131.32, 133.59, 136.7, 137.2, 155.7, 163.4, 166.4; υ_max_ (cm^−1^) 3180, NH stretch, primary amide; 1679, C = O stretch, carboxylic acid; 1679, C = O stretch, primary amide. FAB/NOBA-MS calculated C_12_H_9_N_2_O_3_S (M+H) 374.9206, found 374.9194.

#### 5-Benzyl-2-(2-bromoacetamido)thiazole-4-carboxylic acid (19)

The title compound was obtained as a brown powder (0.3 g, 33.3%) using general procedure C. mp 220–222°C; ^1^H NMR (400 MHz, DMSO-*d*_6_) δ 4.10 (s, 2H), 4.49 (s, 2H), 7.32 (m, 5H), 12.80 (s, 1H); ^13^C NMR (400 MHz, DMSO-*d*_6_) δ 28.7, 32.5, 127.24, 129.08, 129.21, 136.8, 140.3, 142.4, 154.3, 164.0, 166.0; υ_max_ (cm^−1^) 3185, NH stretch, primary amide; 1695, C = O stretch, carboxylic acid; 1661, C = O stretch, primary amide; FTMS-ESI calculated C_13_H_12_N_2_O_3_SBr (M+H) 354.9752, found 354.9736.

#### mtFabH inhibition assay

The condensing activity of mtFabH was assayed by mixing 100 µM holo-AcpM, 1 mM β-mercaptoethanol, 0.1 M sodium phosphate buffer pH 7.0, 50 µM malonyl-CoA, 45 nCi of [2-^14^C]malonyl-CoA (specific activity 53 Ci/mol), 12.5 µM of palmitoyl-CoA, and 0.3 µg mtFabD in a final volume of 40 µl. The mtFabD protein was added to generate the malonyl-AcpM substrate for the reaction *in situ*. A mixture of AcpM, 1 mM β-mercaptoethanol, and the buffer was incubated at 37°C for 30 min to ensure complete reduction of AcpM, and then the remaining components (except mtFabH) were added. The mixture was then dispensed into microcentrifuge tubes along with the test compound (0.5–200 µg/ml), and the reaction was initiated by the addition of 0.5 µg of mtFabH. The reaction mixture was held at 37°C for 40 min. The reaction was quenched by adding 5 mg/ml NaBH_4_ in 100 mM K_2_HPO_4_, 100 mM KCl, 30% tetrahydrofuran, resulting in the liberation of β-ketoacyl groups from their respective thioesters as acyl-1,3-diols. These products were extracted three times with 2 ml of water-saturated toluene, and the extracts were pooled and washed with an equal volume of water. The organic component was transferred to scintillation vials, the solvent was removed by evaporation, 5 ml EcoScintA (National Diagnostics, UK) was added, and the radiolabeled products were quantified by liquid scintillation counting.

#### FAS-I and FAS-II Assays

FAS-I and FAS-II experiments were conducted as *per* Slayden *et al.* (1996) using the 40–80% ammonium sulphate fraction. The standard reaction mixture for the incorporation of radioactivity from [2-^14^C]malonyl-CoA into C_16_ to C_24_ fatty acids catalysed by FAS-I was composed as follows: 100 mM Tris.HCl pH 7.9, 5 mM EDTA, 5 mM dithiothreitol, 300 µM acetyl-CoA, 100 µM NADPH, 100 µM NADH, 1 µM flavin mononucleotide, 500 µM α-cyclodextrin, 300 µM CoA-SH, 100,000 cpm of [2-^14^C] malonyl-CoA (specific activity 53 Ci/mol), test compound (0.5–200 µg/ml) and 2 mg of cytosolic enzyme preparation in a total volume of 500 µl. Similarly, the standard reaction mixture for incorporation of radioactivity from [2-^14^C]malonyl-CoA into C_24_ to C_30_ fatty acids catalysed by FAS-II contained the following: 100 mM potassium phosphate buffer (pH 7.0), 5 mM EDTA, 5 mM dithiothreitol, 10 µM palmitoyl-CoA, 140 µM NADPH, 140 µM NADH, 100 µg of AcpM, 40 µM malonyl-CoA, 200,000 cpm of [2-^14^C]malonyl-CoA (specific activity 53 Ci/mol), test compound (0.5–200 µg/ml) and 200 mg of cytosolic enzyme preparation in a total volume of 250 µl. In both the FAS-I and FAS-II assays, reactions were performed in triplicate at 37°C for 30 min and terminated by the addition of 250 µl of 20% potassium hydroxide in 50% methanol at 100°C for 45 min. Following acidification with 150 µl of 6 M HCl, the resultant ^14^C-labelled fatty acids were extracted three times with petroleum ether. The organic extracts were pooled, washed once with an equal volume of water, and dried in a scintillation vial prior to counting.

#### *M. tuberculosis* H_37_R_v_ inhibition assays

All compounds were screened for their *in vitro* antimycobacterial activity against *M. tuberculosis* H37Rv by an in-house broth macrodilution method. The activity of compounds was confirmed by MIC determination by different methods against *M. tuberculosis* H37Rv, which included: broth microdilution assay, resazurin microdilution assay, agar dilution proportion method and the MB/Bactec 3D system. Positive and negative growth controls were run in each experiment run and ciprofloxacin was used as an antibiotic control (MIC = 0.025–0.5 µg/ml would indicate that the experiment is successful). Unless stated otherwise, the inoculum was 100 µl of 1 McFarland broth during log phase. A 21 gauge fine-needled syringe was used to flush the broth 5 times to break up any clumps. Blood agar plates were inoculated to check contamination. Here is a brief description of each method:

#### Broth macrodilution assay

A stock solution of each compound (1 mg/ml) was diluted in sterile distilled water to test the range (0.06–32 µg/ml). Each tube contained 4 ml sterile Middlebrook 7H9 broth containing albumin-dextrose-catalase (ADC supplement, Oxoid), Tween 80, glycerol and 4 ml of the compound solution was added to make serial double dilutions. Tubes were incubated at 37°C for 7 days and then read visually. MIC was determined as the lowest concentration of antibiotic that prevented turbidity.

#### Resazurin microdilution assay

96-well microtitre plates with U-bottomed wells (Nunclon, Denmark) were used and stock solutions (1 mg/ml) were diluted to four fold the final highest concentration tested [29]. Three ranges were tested in duplicate: 0.2–0.025 µg/ml, 0.3–0.037 µg/ml and 0.5–0.06 µg/ml. A two-fold dilution series was prepared with 100 µl antibiotic solution in sterile distilled water. The microtitre plates were covered with lids and placed inside a safety box (Nalgene, USA) and incubated at 37°C for seven days. On the seventh day, 30 µl resazurin (prepared as 0.01% and store at 4°C until use within one week) was added and incubated at 37°C for another 24 h. Plates were read on the following day and MIC was determined as the lowest concentration of antibiotic that prevented colour change (blue to pink).

#### Microdilution broth assay

Microdilution broth assay was performed simultaneously with the resazurin assay using same method but without the 1∶20 dilution step. The MIC was determined after fourteen days by reading the microtitre plate visually.

#### Agar proportion method

The MIC of each compound was tested by agar dilution in triplicate as recommended by the National Committee for Clinical Laboratory Standards (NCCLS). The compounds dilution range was (0.3–0.025 µg/ml) and stock solutions were diluted to tenfold the final highest concentration tested. Plates were prepared as by adding 2 ml of the compound solution to 18 ml of Middlebrook 7H10 agar containing oleic acid-albumin-dextrose-catalase supplement (OADC, Oxoid), sealed and stored at 4°C to be used within one week of preparation. Each plate was inoculated with 20 µl 1 McFarland broth in the log phase diluted to different concentrations (10^−1^ to 10^−6^ dilutions). Plates were incubated at 37°C for fourteen days. The MIC was considered the lowest concentration that showed no visible colonies at all dilutions.

#### MIC measurement by MB/Bactec 3D system

Stock solutions of the compounds were prepared as 10,000 times the desired concentration and diluted accordingly with the liquid medium provided in the MB bottles. Each bottle was inoculated with 0.5 ml Middlebrook 7H9 broth culture in the log phase with growth of 1.0 McFarland turbidity. A positive (1%) and a negative control and isoniazid control were included. Bottles were kept in the automated system for 56 days. MIC was considered as the lowest concentration of compound that did not grow or gave growth signal later than the (1%) control.

#### HS27 toxicity studies

The HS27 cells (grown in Dulbecco's modified eagle medium, supplemented with FBS, penicillin/streptomycin, *L*-glutamine and sodium pyruvate (Invitrogen) were plated in 96 well plates from Helena Bioscience at 1×10e4 cells per well (80 ul/well) and incubated for 24 hrs at 37°C, 5% CO_2_. The samples (10 ul/well) and alamar blue (10 ul/well) were added, the cells incubated in the presence of sample at 37°C, 5% CO_2_ for 24 hrs before reading in the fluorescence mode (560/590 nm).
